# 1,5-Bis(thio­phen-2-yl)-3-(2,4,5-trimeth­oxy­phen­yl)pentane-1,5-dione

**DOI:** 10.1107/S160053681104846X

**Published:** 2011-11-25

**Authors:** Hoong-Kun Fun, Thitipone Suwunwong, Nawong Boonnak, Suchada Chantrapromma

**Affiliations:** a X-ray Crystallography Unit, School of Physics, Universiti Sains Malaysia, 11800 USM, Penang, Malaysia; b Crystal Materials Research Unit, Department of Chemistry, Faculty of Science, Prince of Songkla University, Hat-Yai, Songkhla 90112, Thailand

## Abstract

In the title 1,5-diketone compound, C_22_H_22_O_5_S_2_, the benzene ring makes dihedral angles of 41.51 (6) and 25.83 (6)° with the two thio­phene rings, while the dihedral angle between the thio­phene rings is 26.67 (7)°. An intra­molecular C—H⋯O inter­action generates an *S*(9) ring motif. In the crystal, mol­ecules are linked into a three-dimensional network by weak C—H⋯O and C—H⋯π inter­actions, and a π–π inter­action with a centroid–centroid distance of 3.6527 (8) Å.

## Related literature

For bond-length data, see: Allen *et al.* (1987 ▶). For hydrogen-bond motifs, see: Bernstein *et al.* (1995 ▶). For background and applications of 1,5-diketone compounds, see: Alagarsamy *et al.* (2007 ▶); Favaro *et al.* (2002 ▶); Harrowven & Hannam (1999 ▶); Pillai *et al.* (2004 ▶); Rai *et al.* (2008 ▶). For the preparation of the title compound, see: Suwunwong *et al.* (2011 ▶). For the stability of the temperature controller used in the data collection, see: Cosier & Glazer (1986 ▶).
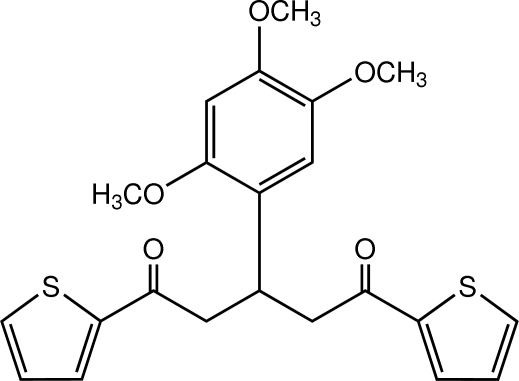


## Experimental

### 

#### Crystal data

C_22_H_22_O_5_S_2_
*M* *_r_* = 430.54
Monoclinic, 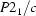

*a* = 16.1955 (2) Å
*b* = 7.5777 (1) Å
*c* = 16.7706 (2) Å
β = 93.490 (1)°
*V* = 2054.35 (4) Å^3^
*Z* = 4
Mo *K*α radiation
μ = 0.29 mm^−1^
*T* = 100 K
0.26 × 0.20 × 0.18 mm

#### Data collection

Bruker APEXII CCD area-detector diffractometer
Absorption correction: multi-scan (*SADABS*; Bruker, 2009 ▶) *T*_min_ = 0.928, *T*_max_ = 0.949
23337 measured reflections
6027 independent reflections
4962 reflections with *I* > 2σ(*I*)
*R*_int_ = 0.030

#### Refinement

*R*[*F* ^2^ > 2σ(*F* ^2^)] = 0.038
*wR*(*F* ^2^) = 0.102
*S* = 1.04
6027 reflections
265 parameters
H-atom parameters constrained
Δρ_max_ = 0.43 e Å^−3^
Δρ_min_ = −0.46 e Å^−3^

### 

Data collection: *APEX2* (Bruker, 2009 ▶); cell refinement: *SAINT* (Bruker, 2009 ▶); data reduction: *SAINT*; program(s) used to solve structure: *SHELXTL* (Sheldrick, 2008 ▶); program(s) used to refine structure: *SHELXTL*; molecular graphics: *SHELXTL*; software used to prepare material for publication: *SHELXTL* and *PLATON* (Spek, 2009 ▶).

## Supplementary Material

Crystal structure: contains datablock(s) global, I. DOI: 10.1107/S160053681104846X/is5007sup1.cif

Structure factors: contains datablock(s) I. DOI: 10.1107/S160053681104846X/is5007Isup2.hkl

Supplementary material file. DOI: 10.1107/S160053681104846X/is5007Isup3.cml

Additional supplementary materials:  crystallographic information; 3D view; checkCIF report

## Figures and Tables

**Table 1:** Hydrogen-bond geometry (Å, °) *Cg*2 is the centroid of the C14–C19 ring.

*D*—H⋯*A*	*D*—H	H⋯*A*	*D*⋯*A*	*D*—H⋯*A*
C2—H2*A*⋯O2^i^	0.95	2.57	3.5002 (18)	166
C3—H3*A*⋯O2	0.95	2.48	3.3677 (17)	156
C8—H8*B*⋯O1^ii^	0.99	2.58	3.3154 (16)	131
C21—H21*A*⋯O1^iii^	0.98	2.57	3.0692 (17)	112
C22—H22*A*⋯O1^iv^	0.98	2.39	3.3489 (16)	165
C21—H21*B*⋯*Cg*2^v^	0.98	2.91	3.8317 (16)	158

Symmetry codes: (i) 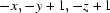; (ii) 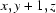; (iii) 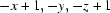; (iv) 
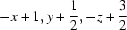
; (v) 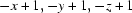.

**Fig. 1.**
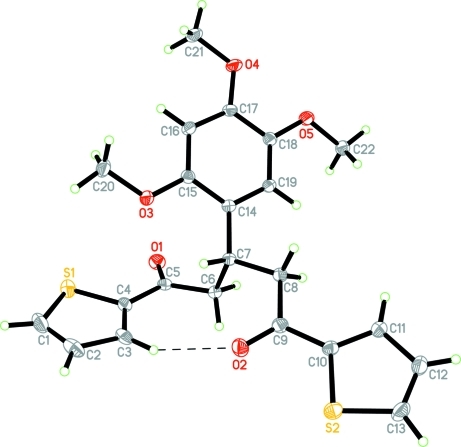
The molecular structure of the title compound, showing 50% probability displacement ellipsoids and the atom-numbering scheme. The weak C—H···O interaction was shown as a dashed line.

**Fig. 2.**
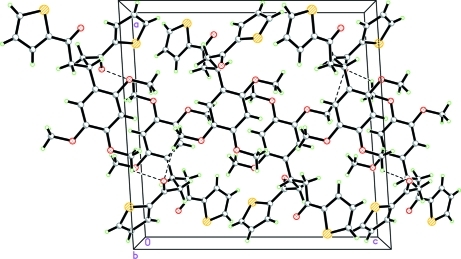
The crystal packing diagram of the title compound viewed along the b axis. Weak C—H···O interactions were shown as dashed lines.

## supplementary crystallographic information

### Comment

Ketones are obviously one of the most important compounds in organic chemistry. There are many interesting applications of the compounds with the ketone group and the heterocyclic unit, which exhibit not only bioactivity such as antibacterial (Rai *et al.*, 2008), anti-inflammatory (Pillai *et al.*, 2004) and analgesic (Alagarsamy *et al.*, 2007) activities, but also fluorescent property (Favaro *et al.*, 2002). The 1,5-diketone compound is conventionally prepared by the oxidative cleavage of cyclopentenes or the conjugate addition of enolates to α,β-unsaturated ketones (Harrowven & Hannam, 1999).

In the molecule of the title compound (Fig. 1), the pentane-1,5-dione unit (C5–C9/O1/O2) is puckered with the torsion angles C5–C6–C7–C8 = 170.36 (10)° and C6–C7–C8–C9 = -71.81 (13)°, making the two ketone groups pointing towards opposite directions. A weak intramolecular C3—H3A···O2 interaction (Table 1) which generates an S(9) ring motif (Bernstein *et al.*, 1995) helps to stabilize this conformation. The dihedral angle between the two thiophene rings is 26.67 (7)°. The 2,4,5-trimethoxyphenyl ring makes dihedral angles of 41.51 (6) and 25.83 (6)° with the S1/C1–C4 and S2/C10–C13 thiophene rings, respectively. The three substituted methoxy groups of 2,4,5-trimethoxyphenyl unit have two different orientations in which the *ortho*- and *para*-methoxy groups (at atom C15 and C17 positions) are co-planar with the phenyl ring with torsion angles C20–O3–C15–C16 = -0.50 (18)° and C21–O4–C17–C18 = 179.46 (12)° whereas the *meta*-methoxy (at atom C18) is twisted with the torsion angle C22–O5–C18–C19 = 10.08 (19)° The bond distances in (I) are in normal ranges (Allen *et al.*, 1987).

In the crystal packing (Fig. 2), the molecules are linked into a three dimensional network through the enone unit by weak C—H···O interactions (Table 1), a weak C—H···π interaction (Table 1) and a π–π interaction with a Cg1···Cg1 distance of 3.6527 (8) Å; Cg1 is the centroid of S1/C1–C4 thiophene ring.

### Experimental

The title compound (I) is a symmetrical 1,5-diketone compound which was alternatively synthesized by the stirring of (*E*)-1-(2-thitenyl)-3-(2,4,5-trimethoxyphenyl)prop-2-en-1-one (0.57 g, 1.5 mmol) (Suwunwong *et al.*, 2011) in methanol (15 ml) with a freshly prepared sodium methoxide (1.5 mmol of sodium in 40 ml of methanol). Excess malononitrile (0.20 g, 3.0 mmol) was then added with continuous stirring at room temperature until the precipitate separated out. The resulting solid was filtered. Colorless single crystals of the title compound suitable for X-ray structure determination was obtained by recrystalization from acetone/methanol (1:1 *v*/*v*) by the slow evaporation of the solvent at room temperature after several days.

### Refinement

All H atoms were placed in calculated positions, with C—H = 0.95, 1.00 and 0.99 Å for aromatic, CH and CH_2_, respectively, and *U*_iso_(H) = 1.2*U*_eq_(C), and with C—H = 0.98 Å and *U*_iso_(H) = 1.5*U*_eq_(C) for CH_3_ atoms. A rotating group model was used for the methyl groups.

### Crystal data

**Table:**

C_22_H_22_O_5_S_2_	*F*(000) = 904
*M**_r_* = 430.54	*D*_x_ = 1.392 Mg m^−^^3^
Monoclinic, *P*2_1_/*c*	Mo *K*α radiation, λ = 0.71073 Å
Hall symbol: -P 2ybc	Cell parameters from 6027 reflections
*a* = 16.1955 (2) Å	θ = 2.4–30.1°
*b* = 7.5777 (1) Å	µ = 0.29 mm^−^^1^
*c* = 16.7706 (2) Å	*T* = 100 K
β = 93.490 (1)°	Block, colorless
*V* = 2054.35 (4) Å^3^	0.26 × 0.20 × 0.18 mm
*Z* = 4	

### Data collection

**Table:**

Bruker APEXII CCD area-detector diffractometer	6027 independent reflections
Radiation source: sealed tube	4962 reflections with *I* > 2σ(*I*)
graphite	*R*_int_ = 0.030
φ and ω scans	θ_max_ = 30.1°, θ_min_ = 2.4°
Absorption correction: multi-scan (*SADABS*; Bruker, 2009)	*h* = −22→22
*T*_min_ = 0.928, *T*_max_ = 0.949	*k* = −10→10
23337 measured reflections	*l* = −23→23

### Refinement

**Table:**

Refinement on *F*^2^	Primary atom site location: structure-invariant direct methods
Least-squares matrix: full	Secondary atom site location: difference Fourier map
*R*[*F*^2^ > 2σ(*F*^2^)] = 0.038	Hydrogen site location: inferred from neighbouring sites
*wR*(*F*^2^) = 0.102	H-atom parameters constrained
*S* = 1.04	*w* = 1/[σ^2^(*F*_o_^2^) + (0.0476*P*)^2^ + 0.7947*P*] where *P* = (*F*_o_^2^ + 2*F*_c_^2^)/3
6027 reflections	(Δ/σ)_max_ = 0.001
265 parameters	Δρ_max_ = 0.43 e Å^−^^3^
0 restraints	Δρ_min_ = −0.46 e Å^−^^3^

### Special details

**Table:**

Experimental. The crystal was placed in the cold stream of an Oxford Cryosystems Cobra open-flow nitrogen cryostat (Cosier & Glazer, 1986) operating at 120.0 (1) K.
Geometry. All esds (except the esd in the dihedral angle between two l.s. planes) are estimated using the full covariance matrix. The cell esds are taken into account individually in the estimation of esds in distances, angles and torsion angles; correlations between esds in cell parameters are only used when they are defined by crystal symmetry. An approximate (isotropic) treatment of cell esds is used for estimating esds involving l.s. planes.
Refinement. Refinement of F^2^ against ALL reflections. The weighted R-factor wR and goodness of fit S are based on F^2^, conventional R-factors R are based on F, with F set to zero for negative F^2^. The threshold expression of F^2^ > 2sigma(F^2^) is used only for calculating R-factors(gt) etc. and is not relevant to the choice of reflections for refinement. R-factors based on F^2^ are statistically about twice as large as those based on F, and R- factors based on ALL data will be even larger.

### Fractional atomic coordinates and isotropic or equivalent isotropic displacement parameters (Å^2^)

**Table:**

	*x*	*y*	*z*	*U*_iso_*/*U*_eq_	
S1	0.15320 (2)	−0.05956 (5)	0.46899 (2)	0.02686 (9)	
S2	0.05511 (2)	0.83742 (5)	0.76099 (2)	0.02604 (9)	
O1	0.26302 (6)	−0.04470 (12)	0.61509 (6)	0.0210 (2)	
O2	0.11405 (6)	0.57870 (14)	0.64643 (6)	0.0245 (2)	
O3	0.31333 (5)	0.28911 (14)	0.48626 (5)	0.0200 (2)	
O4	0.60447 (5)	0.28712 (14)	0.57867 (6)	0.0221 (2)	
O5	0.56590 (6)	0.37664 (15)	0.72042 (6)	0.0228 (2)	
C1	0.07060 (9)	0.0369 (2)	0.41872 (9)	0.0287 (3)	
H1A	0.0469	−0.0046	0.3690	0.034*	
C2	0.04213 (9)	0.1804 (2)	0.45787 (9)	0.0261 (3)	
H2A	−0.0042	0.2484	0.4388	0.031*	
C3	0.08930 (8)	0.21756 (18)	0.53040 (8)	0.0203 (3)	
H3A	0.0790	0.3136	0.5648	0.024*	
C4	0.15231 (8)	0.09482 (17)	0.54423 (8)	0.0171 (2)	
C5	0.21609 (7)	0.08266 (16)	0.60996 (7)	0.0154 (2)	
C6	0.22329 (7)	0.23228 (17)	0.66977 (7)	0.0158 (2)	
H6A	0.2561	0.1920	0.7181	0.019*	
H6B	0.1674	0.2641	0.6858	0.019*	
C7	0.26495 (7)	0.39825 (16)	0.63566 (7)	0.0144 (2)	
H7A	0.2345	0.4293	0.5839	0.017*	
C8	0.25704 (7)	0.55376 (17)	0.69304 (8)	0.0169 (2)	
H8A	0.2753	0.5152	0.7477	0.020*	
H8B	0.2946	0.6493	0.6777	0.020*	
C9	0.16998 (8)	0.62670 (17)	0.69406 (8)	0.0176 (2)	
C10	0.15454 (8)	0.75825 (17)	0.75553 (8)	0.0172 (2)	
C11	0.20817 (8)	0.83333 (17)	0.81330 (8)	0.0184 (2)	
H11A	0.2656	0.8079	0.8194	0.022*	
C12	0.16658 (9)	0.95354 (18)	0.86262 (8)	0.0229 (3)	
H12A	0.1931	1.0169	0.9059	0.027*	
C13	0.08427 (9)	0.96710 (19)	0.84048 (9)	0.0269 (3)	
H13A	0.0472	1.0412	0.8668	0.032*	
C14	0.35481 (7)	0.36293 (16)	0.61902 (7)	0.0141 (2)	
C15	0.37699 (7)	0.31052 (16)	0.54348 (7)	0.0152 (2)	
C16	0.46025 (7)	0.28376 (17)	0.52796 (7)	0.0164 (2)	
H16A	0.4744	0.2485	0.4762	0.020*	
C17	0.52174 (7)	0.30865 (17)	0.58790 (8)	0.0163 (2)	
C18	0.50086 (7)	0.35820 (17)	0.66468 (7)	0.0161 (2)	
C19	0.41810 (7)	0.38428 (17)	0.67889 (7)	0.0155 (2)	
H19A	0.4040	0.4177	0.7309	0.019*	
C20	0.33368 (9)	0.2389 (2)	0.40790 (8)	0.0235 (3)	
H20A	0.2827	0.2213	0.3742	0.035*	
H20B	0.3655	0.1288	0.4107	0.035*	
H20C	0.3668	0.3320	0.3849	0.035*	
C21	0.62830 (8)	0.2351 (2)	0.50147 (8)	0.0224 (3)	
H21A	0.6888	0.2286	0.5018	0.034*	
H21B	0.6079	0.3219	0.4617	0.034*	
H21C	0.6046	0.1191	0.4880	0.034*	
C22	0.54575 (8)	0.39764 (19)	0.80143 (8)	0.0205 (3)	
H22A	0.5967	0.4118	0.8354	0.031*	
H22B	0.5158	0.2931	0.8184	0.031*	
H22C	0.5109	0.5024	0.8061	0.031*	

### Atomic displacement parameters (Å^2^)

**Table:**

	*U*^11^	*U*^22^	*U*^33^	*U*^12^	*U*^13^	*U*^23^
S1	0.02481 (18)	0.0312 (2)	0.02430 (18)	−0.00215 (14)	−0.00106 (13)	−0.00875 (14)
S2	0.01850 (16)	0.02694 (18)	0.0325 (2)	0.00698 (13)	−0.00001 (13)	−0.00871 (14)
O1	0.0187 (4)	0.0189 (5)	0.0252 (5)	0.0036 (4)	0.0008 (4)	−0.0002 (4)
O2	0.0178 (4)	0.0264 (5)	0.0284 (5)	0.0055 (4)	−0.0049 (4)	−0.0080 (4)
O3	0.0151 (4)	0.0313 (5)	0.0136 (4)	0.0000 (4)	0.0000 (3)	−0.0038 (4)
O4	0.0122 (4)	0.0352 (6)	0.0194 (5)	0.0024 (4)	0.0040 (3)	−0.0034 (4)
O5	0.0134 (4)	0.0392 (6)	0.0156 (4)	0.0004 (4)	−0.0007 (3)	−0.0041 (4)
C1	0.0254 (7)	0.0389 (9)	0.0209 (7)	−0.0112 (6)	−0.0061 (5)	0.0037 (6)
C2	0.0195 (6)	0.0287 (7)	0.0292 (7)	−0.0066 (5)	−0.0054 (5)	0.0131 (6)
C3	0.0166 (6)	0.0212 (6)	0.0227 (6)	−0.0059 (5)	−0.0017 (5)	0.0037 (5)
C4	0.0155 (5)	0.0176 (6)	0.0183 (6)	−0.0029 (4)	0.0008 (4)	−0.0005 (5)
C5	0.0130 (5)	0.0167 (6)	0.0168 (6)	−0.0014 (4)	0.0031 (4)	0.0008 (4)
C6	0.0143 (5)	0.0171 (6)	0.0162 (6)	−0.0001 (4)	0.0023 (4)	−0.0006 (4)
C7	0.0124 (5)	0.0155 (5)	0.0152 (5)	0.0008 (4)	0.0011 (4)	−0.0006 (4)
C8	0.0131 (5)	0.0180 (6)	0.0194 (6)	0.0017 (4)	0.0002 (4)	−0.0030 (5)
C9	0.0162 (6)	0.0167 (6)	0.0199 (6)	0.0024 (4)	0.0007 (5)	−0.0004 (5)
C10	0.0154 (5)	0.0169 (6)	0.0195 (6)	0.0023 (4)	0.0022 (4)	0.0002 (5)
C11	0.0193 (6)	0.0177 (6)	0.0183 (6)	0.0009 (5)	0.0023 (5)	−0.0007 (5)
C12	0.0273 (7)	0.0214 (6)	0.0202 (6)	−0.0016 (5)	0.0029 (5)	−0.0034 (5)
C13	0.0291 (7)	0.0223 (7)	0.0300 (7)	0.0041 (6)	0.0082 (6)	−0.0074 (6)
C14	0.0129 (5)	0.0146 (5)	0.0149 (5)	0.0003 (4)	0.0021 (4)	0.0005 (4)
C15	0.0145 (5)	0.0169 (6)	0.0141 (5)	−0.0007 (4)	−0.0001 (4)	0.0011 (4)
C16	0.0163 (5)	0.0188 (6)	0.0146 (6)	0.0007 (4)	0.0038 (4)	−0.0003 (4)
C17	0.0127 (5)	0.0189 (6)	0.0174 (6)	0.0006 (4)	0.0030 (4)	0.0004 (5)
C18	0.0138 (5)	0.0191 (6)	0.0152 (6)	0.0002 (4)	0.0000 (4)	0.0000 (4)
C19	0.0149 (5)	0.0175 (6)	0.0144 (5)	0.0004 (4)	0.0020 (4)	−0.0008 (4)
C20	0.0236 (6)	0.0320 (7)	0.0148 (6)	0.0027 (6)	−0.0004 (5)	−0.0045 (5)
C21	0.0189 (6)	0.0279 (7)	0.0212 (6)	0.0034 (5)	0.0072 (5)	−0.0021 (5)
C22	0.0178 (6)	0.0279 (7)	0.0156 (6)	0.0006 (5)	−0.0008 (5)	−0.0024 (5)

### Geometric parameters (Å, °)

**Table:**

S1—C1	1.7021 (16)	C8—H8A	0.9900
S1—C4	1.7214 (13)	C8—H8B	0.9900
S2—C13	1.6998 (15)	C9—C10	1.4665 (18)
S2—C10	1.7261 (13)	C10—C11	1.3835 (19)
O1—C5	1.2283 (15)	C11—C12	1.4266 (18)
O2—C9	1.2259 (16)	C11—H11A	0.9500
O3—C15	1.3747 (15)	C12—C13	1.366 (2)
O3—C20	1.4258 (15)	C12—H12A	0.9500
O4—C17	1.3678 (14)	C13—H13A	0.9500
O4—C21	1.4288 (16)	C14—C15	1.3954 (16)
O5—C18	1.3722 (15)	C14—C19	1.3998 (17)
O5—C22	1.4253 (15)	C15—C16	1.4035 (16)
C1—C2	1.365 (2)	C16—C17	1.3842 (18)
C1—H1A	0.9500	C16—H16A	0.9500
C2—C3	1.425 (2)	C17—C18	1.4022 (17)
C2—H2A	0.9500	C18—C19	1.3897 (16)
C3—C4	1.3897 (19)	C19—H19A	0.9500
C3—H3A	0.9500	C20—H20A	0.9800
C4—C5	1.4669 (18)	C20—H20B	0.9800
C5—C6	1.5136 (17)	C20—H20C	0.9800
C6—C7	1.5527 (17)	C21—H21A	0.9800
C6—H6A	0.9900	C21—H21B	0.9800
C6—H6B	0.9900	C21—H21C	0.9800
C7—C14	1.5222 (16)	C22—H22A	0.9800
C7—C8	1.5317 (17)	C22—H22B	0.9800
C7—H7A	1.0000	C22—H22C	0.9800
C8—C9	1.5155 (17)		
			
C1—S1—C4	91.71 (7)	C10—C11—H11A	124.1
C13—S2—C10	91.55 (7)	C12—C11—H11A	124.1
C15—O3—C20	117.99 (10)	C13—C12—C11	112.20 (13)
C17—O4—C21	117.13 (10)	C13—C12—H12A	123.9
C18—O5—C22	116.74 (10)	C11—C12—H12A	123.9
C2—C1—S1	112.47 (11)	C12—C13—S2	112.93 (11)
C2—C1—H1A	123.8	C12—C13—H13A	123.5
S1—C1—H1A	123.8	S2—C13—H13A	123.5
C1—C2—C3	112.88 (13)	C15—C14—C19	117.79 (11)
C1—C2—H2A	123.6	C15—C14—C7	121.22 (11)
C3—C2—H2A	123.6	C19—C14—C7	120.98 (10)
C4—C3—C2	111.13 (13)	O3—C15—C14	116.35 (10)
C4—C3—H3A	124.4	O3—C15—C16	122.80 (11)
C2—C3—H3A	124.4	C14—C15—C16	120.85 (11)
C3—C4—C5	130.07 (12)	C17—C16—C15	120.17 (11)
C3—C4—S1	111.80 (10)	C17—C16—H16A	119.9
C5—C4—S1	118.11 (9)	C15—C16—H16A	119.9
O1—C5—C4	120.42 (12)	O4—C17—C16	124.60 (11)
O1—C5—C6	121.32 (12)	O4—C17—C18	115.38 (11)
C4—C5—C6	118.25 (11)	C16—C17—C18	120.02 (11)
C5—C6—C7	112.35 (10)	O5—C18—C19	125.25 (11)
C5—C6—H6A	109.1	O5—C18—C17	115.77 (10)
C7—C6—H6A	109.1	C19—C18—C17	118.98 (11)
C5—C6—H6B	109.1	C18—C19—C14	122.17 (11)
C7—C6—H6B	109.1	C18—C19—H19A	118.9
H6A—C6—H6B	107.9	C14—C19—H19A	118.9
C14—C7—C8	111.59 (10)	O3—C20—H20A	109.5
C14—C7—C6	111.52 (10)	O3—C20—H20B	109.5
C8—C7—C6	109.69 (10)	H20A—C20—H20B	109.5
C14—C7—H7A	108.0	O3—C20—H20C	109.5
C8—C7—H7A	108.0	H20A—C20—H20C	109.5
C6—C7—H7A	108.0	H20B—C20—H20C	109.5
C9—C8—C7	113.65 (10)	O4—C21—H21A	109.5
C9—C8—H8A	108.8	O4—C21—H21B	109.5
C7—C8—H8A	108.8	H21A—C21—H21B	109.5
C9—C8—H8B	108.8	O4—C21—H21C	109.5
C7—C8—H8B	108.8	H21A—C21—H21C	109.5
H8A—C8—H8B	107.7	H21B—C21—H21C	109.5
O2—C9—C10	120.59 (11)	O5—C22—H22A	109.5
O2—C9—C8	122.38 (12)	O5—C22—H22B	109.5
C10—C9—C8	117.03 (11)	H22A—C22—H22B	109.5
C11—C10—C9	130.18 (11)	O5—C22—H22C	109.5
C11—C10—S2	111.58 (9)	H22A—C22—H22C	109.5
C9—C10—S2	118.23 (10)	H22B—C22—H22C	109.5
C10—C11—C12	111.72 (12)		
			
C4—S1—C1—C2	−0.79 (12)	C11—C12—C13—S2	0.05 (17)
S1—C1—C2—C3	1.29 (16)	C10—S2—C13—C12	0.45 (12)
C1—C2—C3—C4	−1.21 (17)	C8—C7—C14—C15	−142.95 (12)
C2—C3—C4—C5	178.95 (12)	C6—C7—C14—C15	93.99 (14)
C2—C3—C4—S1	0.60 (14)	C8—C7—C14—C19	36.21 (16)
C1—S1—C4—C3	0.09 (10)	C6—C7—C14—C19	−86.85 (14)
C1—S1—C4—C5	−178.48 (10)	C20—O3—C15—C14	178.82 (12)
C3—C4—C5—O1	174.25 (13)	C20—O3—C15—C16	−0.50 (18)
S1—C4—C5—O1	−7.49 (16)	C19—C14—C15—O3	179.39 (11)
C3—C4—C5—C6	−6.94 (19)	C7—C14—C15—O3	−1.42 (17)
S1—C4—C5—C6	171.32 (9)	C19—C14—C15—C16	−1.26 (18)
O1—C5—C6—C7	103.44 (13)	C7—C14—C15—C16	177.92 (11)
C4—C5—C6—C7	−75.37 (13)	O3—C15—C16—C17	179.52 (11)
C5—C6—C7—C14	−65.51 (13)	C14—C15—C16—C17	0.22 (19)
C5—C6—C7—C8	170.36 (10)	C21—O4—C17—C16	−0.06 (19)
C14—C7—C8—C9	164.10 (10)	C21—O4—C17—C18	179.46 (12)
C6—C7—C8—C9	−71.81 (13)	C15—C16—C17—O4	−179.56 (12)
C7—C8—C9—O2	−8.19 (18)	C15—C16—C17—C18	0.94 (19)
C7—C8—C9—C10	171.37 (11)	C22—O5—C18—C19	10.08 (19)
O2—C9—C10—C11	−178.32 (14)	C22—O5—C18—C17	−169.80 (12)
C8—C9—C10—C11	2.1 (2)	O4—C17—C18—O5	−0.66 (17)
O2—C9—C10—S2	2.17 (18)	C16—C17—C18—O5	178.88 (12)
C8—C9—C10—S2	−177.40 (9)	O4—C17—C18—C19	179.45 (11)
C13—S2—C10—C11	−0.83 (11)	C16—C17—C18—C19	−1.01 (19)
C13—S2—C10—C9	178.76 (11)	O5—C18—C19—C14	−179.96 (12)
C9—C10—C11—C12	−178.53 (13)	C17—C18—C19—C14	−0.08 (19)
S2—C10—C11—C12	1.00 (15)	C15—C14—C19—C18	1.20 (19)
C10—C11—C12—C13	−0.68 (17)	C7—C14—C19—C18	−177.99 (11)

### Hydrogen-bond geometry (Å, °)

**Table:**

*Cg*2 is the centroid of the C14–C19 ring.

**Table:**

*D*—H···*A*	*D*—H	H···*A*	*D*···*A*	*D*—H···*A*
C2—H2A···O2^i^	0.95	2.57	3.5002 (18)	166
C3—H3A···O2	0.95	2.48	3.3677 (17)	156
C8—H8B···O1^ii^	0.99	2.58	3.3154 (16)	131
C21—H21A···O1^iii^	0.98	2.57	3.0692 (17)	112
C22—H22A···O1^iv^	0.98	2.39	3.3489 (16)	165
C21—H21B···Cg2^v^	0.98	2.91	3.8317 (16)	158

Symmetry codes: (i) −*x*, −*y*+1, −*z*+1;  (ii) *x*, *y*+1, *z*; (iii) −*x*+1, −*y*, −*z*+1; (iv) −*x*+1, *y*+1/2, −*z*+3/2; (v) −*x*+1, −*y*+1, −*z*+1.

## References

[bb1] Alagarsamy, V., Vijayakumar, S. & Solomon, V. R. (2007). *Biomed. Pharmacother.* **61**, 285–291. 10.1016/j.biopha.2007.02.008 17391907

[bb2] Allen, F. H., Kennard, O., Watson, D. G., Brammer, L., Orpen, A. G. & Taylor, R. (1987). *J. Chem. Soc. Perkin Trans. 2*, pp. S1–19.

[bb3] Bernstein, J., Davis, R. E., Shimoni, L. & Chang, N.-L. (1995). *Angew. Chem. Int. Ed. Engl.* **34**, 1555–1573.

[bb4] Bruker (2009). *APEX2*, *SAINT* and *SADABS* Bruker AXS Inc., Madison, Wisconsin, USA.

[bb5] Cosier, J. & Glazer, A. M. (1986). *J. Appl. Cryst.* **19**, 105–107.

[bb6] Favaro, G., Ortica, F. & Romani, A. (2002). *Chem. Phys.* **280**, 163–175.

[bb7] Harrowven, D. C. & Hannam, J. C. (1999). *Tetrahedron*, **55**, 9333–9340.

[bb8] Pillai, A. D., Rathod, P. D., Franklin, P. X., Padh, H., Vasu, K. K. & Sudarsanam, V. (2004). *Biochem. Biophys. Res. Commun.* **317**, 1067–1074. 10.1016/j.bbrc.2004.03.148 15094377

[bb9] Rai, N. S., Kalluraya, B., Lingappa, B., Shenoy, S. & Puranic, V. G. (2008). *Eur. J. Med. Chem.* **43**, 1715–1720. 10.1016/j.ejmech.2007.08.002 17923171

[bb10] Sheldrick, G. M. (2008). *Acta Cryst.* A**64**, 112–122. 10.1107/S0108767307043930 18156677

[bb11] Spek, A. L. (2009). *Acta Cryst.* D**65**, 148–155. 10.1107/S090744490804362X PMC2631630 19171970

[bb12] Suwunwong, T., Chantrapromma, S. & Fun, H.-K. (2011). *Chem. Zvesti*, **65**, 890–897.

